# Comparison of coronary endothelial function and brachial flow mediated vasodilatation using cardiac magnetic resonance imaging

**DOI:** 10.1186/1532-429X-11-S1-P35

**Published:** 2009-01-28

**Authors:** Dipti Gupta, Lynette Duncanson, Danielle Janosevic, William Schapiro, Jing Han, Katherine McGrath, Nathaniel Reichek

**Affiliations:** grid.416387.f St. Francis Hospital, Roslyn, NY USA

**Keywords:** Left Anterior Descend, Cold Pressor Testing, Coronary Risk Factor, Flow Mediate Vasodilatation, Coronary Endothelial Function

## Introduction

Brachial artery flow-mediated dilatation (FMD) is used as a surrogate for invasive assessment of coronary artery endothelial function, concordance with invasive assessment of coronary endothelial function is controversial.

## Purpose

We assessed both brachial FMD and coronary CPT noninvasively at the same sitting using cardiac MRI (CMR) and compared the two in a population with and without coronary risk factors.

## Methods

Endothelium-dependent vasodilator function was assessed in the brachial and coronary arteries in 39 subjects. Using a Siemens 1.5 T scanner, the left anterior descending (LAD) coronary artery was localized using a navigator angiographic sequence and a proximal cross-section imaged using TrueFISP bright blood imaging and T2 weighted turbo spin echo imaging. Images were obtained at rest and during cold pressor testing(CPT) and evaluated in ARGUS (Siemens). The % change in lumen cross-sectional area in mid diastole was determined. FMD was assessed by inflating a blood pressure cuff to a reading 50 mmHg higher than the subject's resting systolic pressure, and deflating after 1 min. Imaging was done using TrueFISP cine bright blood imaging at rest, inflation and at 5 sequential time points during deflation. Images were evaluated in ARGUS (Siemens). The % change in lumen cross-sectional area pre and post inflation was determined.

## Results

Of 39 subjects, 16 were females and the mean age was 52 ± 8 yrs. Coronary risk factors (hypertension, hyperlipidemia, smoking, diabetes) were present in 19 while 20 had no risk factors.

The mean brachial FMD of the pooled cohort was 13% and the mean coronary response to CPT was 16%. There was no significant relationship between the brachial FMD and coronary CPT response (r = 0.03, P = 0.8).

## Conclusion

When determined with similar noninvasive methods at the same sitting, brachial FMD does not correlate closely with coronary endothelial response to CPT in a population with and without coronary risk factors. Use of brachial FMD as a surrogate for coronary endothelial function should be reconsidered.

